# Management of oesophageal foreign bodies in children: a 10-year retrospective analysis from a tertiary care center

**DOI:** 10.1186/s12873-022-00723-4

**Published:** 2022-10-04

**Authors:** Guo Xu, Yong-chao Chen, Jing Chen, De-sheng Jia, Ze-bin Wu, Lan Li

**Affiliations:** 1 Department of Otorhinolaryngology, Shenzhen Children’s Hospital, 7019 Yitian Road, Futian District, Shenzhen, 518038 Guangdong China; 2 Department of Otorhinolaryngology, Shenzhen Children’s Hospital, China Medical University, Shenzhen, 518038 Guangdong China

**Keywords:** Oesophageal foreign body, Children, Foley catheter, Rigid oesophageal endoscopy

## Abstract

**Objective:**

Oesophageal foreign bodies (EFBs) are a common emergency issue in paediatrics, and few studies have revealed its clinical features and treatment methods. We conducted this retrospective study to provide our 10-year clinical evidence for the diagnosis and treatment of EFB and reduce the incidence of complications.

**Methods:**

We retrospectively reviewed all paediatric cases diagnosed with EFB from January 2012 to December 2021 at Shenzhen Children’s Hospital. The age and sex of the patients, types of foreign bodies (FBs), preoperative examination, location and duration of FB impaction, clinical symptoms, surgical methods, therapeutic effects and complications were analysed.

**Results:**

Among the 1355 cases, 759 were boys and 596 were girls, with a median age of 2.9 years (4 months to 16 years). The shortest FB lodged time was 1 hour, while the longest time was 3 months. The types of foreign bodies included coins and blunt objects (812,59.9%), bones and sharp objects (278,20.5%), button batteries (86,6.3%), food impactions (84,6.2%), toys (51,3.8%) and plastic objects (44,3.2%). A total of 720 of 812 cases impacted by coins and blunt subjects were successfully treated with a Foley catheter without any complications. A total of 558 patients underwent rigid oesophageal endoscopy under general anaesthesia, and foreign bodies were successfully removed in 525 cases. No FB was found in 33 cases, and FBs pushed into the lower digestive tract during operation in 5 cases. Oesophageal injury was found in 130 cases (23.3%). Our study showed that the age of the patient, time of foreign body incarceration, type of foreign body, location of the lodged foreign body, and fever or cough were risk factors leading to oesophageal foreign body complications, and the differences were statistically significant (*P* < 0.05).

**Conclusion:**

Children with EFB have a risk of complications, especially if the FB is a button battery. The appropriate surgical method should be selected through the analysis of the clinical characteristics of the foreign body in the oesophagus and the risk factors for complications to reduce the incidence of complications. Health education and effective care are the keys to the prevention of EFB.

## Introduction

Oesophageal foreign bodies (EFBs) are a very common emergency for otolaryngologists and often require urgent treatment. Patients usually present with dysphagia, vomiting, drooling, feeding refusal, neck and chest pain, and large FBs even compressing the airway may present with stridor, wheezing, and respiratory distress. EFBs are particularly common in children and is mainly detected in children less than 5 years of age because young children are prone to putting objects in their mouths and swallowing them [1–3]. Orsagh [2] showed that foreign body ingestion (FBI) had a prevalence of up to 17.9 per 10,000 children in U.S. emergency departments in 2015. The most common FB is coins, and FBs mostly do not induce serious complications. However, with changes in the types of FB, such as big toys, batteries, high-powered magnet ingestions, and various other objects that may be sharp or blunt and vary in size, the complications and the risk of death increase among children [4]. Complaints of EFBs are often absent in children compared with adults, which may lead to delays in diagnosis and treatment and subsequent complications such as oesophageal perforation, periesophagitis, and mediastinal infection. It is reported in the literature that approximately 1500 people die each year due to EFBs [5]. Many EFBs pass on their own, but often the easiest and least anxiety-producing decision is to proceed to endoscopic removal among children instead of observation alone [6]. The most common treatment strategies for treating EFBs in children include rigid oesophagoscopy and Foley catheter balloon extraction, among others [6]. The choice of treatment modality depends on the characteristics of the foreign body, its composition, size, location, and presenting symptoms. Each technique has its own unique set of risks and benefits. However, overall data on the adverse events associated with EFBs and the management techniques for EFBs are still limited. In this study, we retrospectively reviewed all paediatric cases of EFB who underwent either rigid endoscopic or Foley catheter balloon removal of FBs over a 10-year period in our department. Revealing the key characteristics and outcomes may be helpful to improving the diagnosis and treatment procedures and reducing the incidence of complications.

## Patients and methods

### Patients and data collection

We retrospectively reviewed all paediatric cases diagnosed with EFB that underwent either rigid endoscopic or Foley catheter balloon removal from January 2012 to December 2021 at Shenzhen Children’s Hospital. We excluded cases with FBs inhaled into the respiratory tract and suspected FB ingestion and cases where imaging or endoscopy revealed no evidence of FBs in the oesophagus.

The included children’s clinical histories were investigated retrospectively. The clinical data collected included demographic data, presenting symptoms, types of foreign bodies, preoperative examination, location and duration of FB impaction, surgical methods, therapeutic effects, complications, and outcomes. For those with more than one admission due to EFBs, only the first admission was recorded. This study was approved by the ethics committee of Shenzhen Children’s Hospital in accordance with the 1964 Helsinki Declaration and its later amendments or comparable ethical standards.

### Treatment strategies for EFBs

Treatment strategies for EFBs require familiarity and skill in the use of different extraction techniques. Every department prefers its own technique for removal, and we usually choose the Foley catheter balloon and rigid endoscopy.

**Fig. 1:**
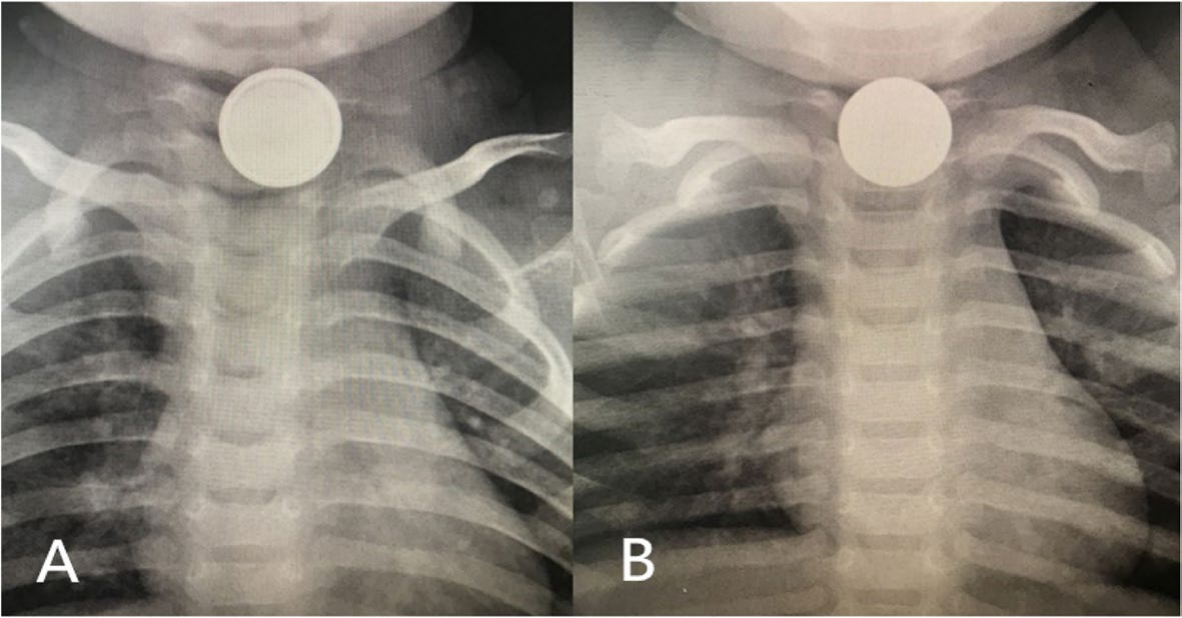
**A** Double ring sign shown in the button battery. **B** The coin in X-ray

Children with witnessed or suspected metal material ingestion need anteroposterior and lateral film X-rays of the chest and abdomen (Fig. 1), while other cases with nonmetallic FBs may receive cervicothoracic CT examination [7]. Cases with obstruction by coins or blunt objects were treated with a Foley catheter in the outpatient department without anaesthesia. Foley catheters with sizes 10–16 (insert inner core) are usually chosen. The children are carefully informed to cooperate by opening the mouth, swallowing, and spitting out the FB in time; any children who were noncooperative were restrained to reduce movement, and a dental pad was placed between the upper and lower teeth to prevent tongue biting. During the procedure for removal of the foreign body, the length of the catheter required to pass beyond the obstruction was roughly measured on the external chest and neck according to the site of the foreign body. The Foley catheter was inserted through the oropharynx into the oesophagus, and care was taken to ensure that it was not coiled in the back of the mouth. The catheter was then 2 to 5 cm deep or fully inserted after reaching the estimated length to ensure that the balloon had been past the FB. After injecting 5 ml of normal saline into the balloon, the catheter was pulled back slowly. Once the object reaches the posterior pharynx, the patient is turned from a prone position to a right decubitus position and allowed to expectorate the object. The resistance increased slightly when passing through the first oesophageal stenosis. Then, the catheter could be pulled out, followed by FB removal into the mouth or direct spitting out. The normal saline for injection could be gradually increased to 10 ml if the FB was not removed the first time.

Rigid oesophageal endoscopy under general anaesthesia with tracheal intubation was performed if the Foley catheter failed three times or the FB was deemed unsafe based on the size and shape. Respiratory distress and intolerable pain during the Foley catheter operation were also indications for endoscopy. The instruments that we used to remove the FB included grasping forceps, rat-toothed forceps, tripod forceps, hollow alligator forceps, and a retrieval basket. The timing of endoscopy was based on the clinical condition of the child and currently available guidelines [6, 8]. Urgent endoscopy was performed within the first 24 hours after the patient was diagnosed with an EFB. A first-aid fast track channel was performed within half an hour when the patient was highly suspected of having a dangerous FB, such as a blade or button battery. When FB removal is difficult and the FB is a coin or blunt object, the FB can be pushed into the stomach, and the FB can complete a safe passage through the intestines. The specifications of endoscopy we used were those of Karl Storz (< 2 years 0.6*1 cm, 3–5 years 0.7*1.0 cm, 6–10 years 0.8*1.1 cm, > 11 years 0.9*1.3 cm).

### Complications

The definition of complications based on oesophagoscopy and imaging performance, damage to the oesophageal mucosa and bleeding were classified as mucosal injury, a mucosal muscularis defect was classified as ulceration, and a submucosal defect was classified as erosion. The diagnoses of periesophagitis, periesophageal abscess, and perforation of the oesophagus were based on the presentation of a piercing foreign body or purulent secretion overflow by oesophageal endoscopy. Oesophageal stenosis, oesophageal perforations, tracheoesophageal fistulas, and periesophageal abscesses may also be found in imaging examinations.

### Statistical analysis

Categorical variables are expressed as numbers and percentages. Continuous variables are reported as the mean ± standard deviation. Comparisons between groups of categorical variables were performed using the χ2 test or Fisher’s exact test. Logistic regression was performed to analyse the risk factors for complications related to EFBs. A *p* value < 0.05 was considered statistically significant. All data were analysed using the statistical package SPSS version 26.0.

## Results

### Demographic characteristics

A total of 1355 cases of EFB were enrolled in the present study; 759 (56%) were boys, and 596 (44%) were girls, with a median age of 2.9 years (4 months to 16 years). Among them, 9 children had hypophrenia, such as Down’ syndrome and autism. Data regarding the characteristics of EFB cases are summarized in Table 1. We divided the age at presentation into 3 groups: most patients were 0–3 years of age (762 cases, 56.2%), followed by the 3–6 years (369 cases, 27.2%) and ≥ 6 years (224 cases, 16.5%).

**Table 1:** Demographic information, patient characteristics and analysis of complications

Patient characteristics	Numbers (%)	Removal methods (RE)^a^	Complications^a^		χ2	***P***
			Yes (***n*** = 130)	No. (***n*** = 428)		
Gender						
Male	759 (56.0%)	308	71	237	0.023	0.879
Female	596 (44.0%)	250	59	191		
Age						
0–3 y	762 (56.2%)	274	84	190	16.376	0.000
3–6 y	369 (27.2%)	135	21	114		
≥ 6y	224 (16.5%)	149	25	124		
Duration						
<24 h	1036 (76.5%)	403	73	330	21.813	0.000
≥ 24 h	319 (23.5%)	155	57	98		
Location						
Upper oesophagus	1120 (82.6%)	399	109	290	12.719	0.002
Middle oesophagus	167 (12.3%)	125	17	108		
Lower oesophagus	68 (5.0%)	34	4	30		
Type						
Blunt object	812 (59.9%)	15	1	14	339.288	0.000
Sharp object	278 (20.5%)	278	35	243		
Button battery	86 (6.3%)	86	86	0		
Food impaction	84 (6.2%)	84	3	81		
Toy	51 (3.8%)	51	4	47		
Plastic	44 (3.2%)	44	1	43		
Symptoms						
Drooling	751 (55.4%)	288	76	212	46.649	0.000
Dysphagia	549 (40.5%)	211	55	156		
Pain	487 (35.9%)	206	43	163		
Vomiting	426 (31.4%)	179	36	143		
Crying	616 (45.5%)	233	39	194		
Cough	101 (7.5%)	32	14	18		
Fever	56 (4.1%)	47	19	28		
Haematemesis	19 (1.4%)	7	7	0		

Upper oesophagus: above the level of T2; middle oesophagus: the level of T3-T6; lower oesophagus: below the level of T7

RE Rigid endoscopy

^a^Only EFB patients who underwent rigid oesophageal endoscopy were analysed

**Fig. 2:**
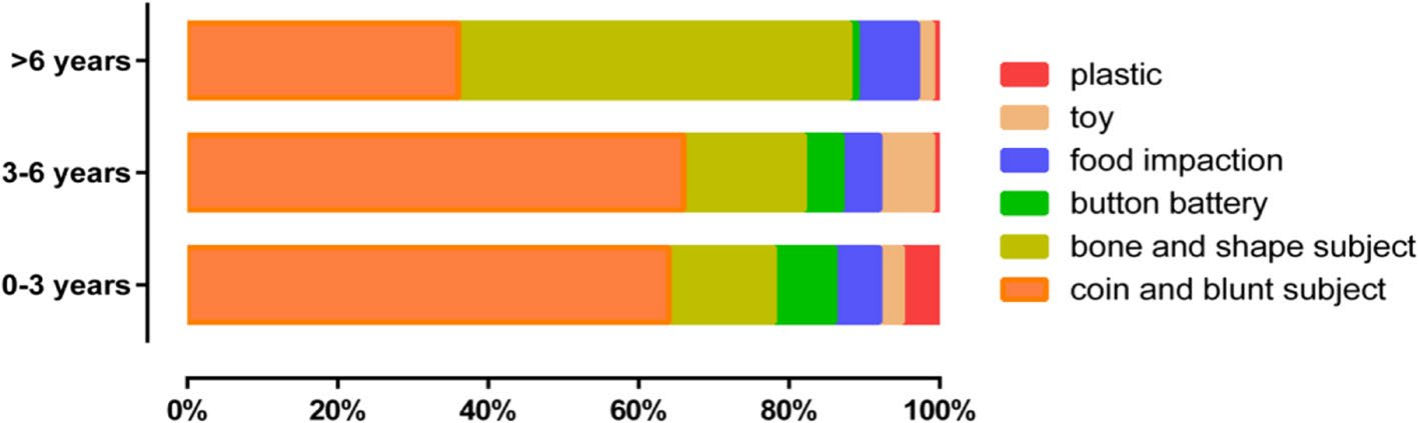
The distribution of FB types differed according to age

Of 1355 patients, 1036 (76.5%) patients sought medical attention within 24 hours, and the remaining 319 (23.5%) patients sought medical attention after 24 hours. The upper oesophagus (above the T2 level) was the most common site of impaction (1120 cases, 82.6%). Blunt subjects (59.9%, including coins, chess pieces, school crests, and buttons) and sharp objects (20.5%, including bones, date pits, glasses, nails, shells, and pins) were the most common FBs in the oesophagus, followed by button batteries (6.3%), food impactions (6.2%), toys (3.8%) and plastics (3.2%). Fig. 2 illustrates that the distribution of FB types differed according to age. Coins and blunt subjects were the most common oesophageal FBs, especially in patients ≤6 years of age.

A history of witnessed FB ingestion was recorded in 82.6% of cases, and other cases were suspected from symptoms. The majority of patients were symptomatic when diagnosed (1278, 94.3%). Patients presented with multiple symptoms, and the most common discomfort was drooling (55.4%). In addition, dysphagia (40.5%), pain (35.9%), vomiting (31.4%), unexplained crying (23.3%), cough (7.5%), fever (4.1%) and haematemesis (1.4%) were observed.

### Treatment outcomes

A total of 720 of 812 cases who were impacted with coin and blunt subjects were successfully treated with a Foley catheter without any complications. Of the remaining 92 cases, 77 FBs entered the gastrointestinal tract (all of which passed without intervention) and were excreted with the stool; meanwhile, the other 15 cases, which included 6 multiple coins, 4 with a presentation time over 1 week, and 3 beads and 2 rings accepted rigid oesophageal endoscopy. No complications, such as oesophageal perforation, infection, or acute airway obstruction, were observed in any of the cases. Two children bit their lips due to struggling.

**Fig. 3:**
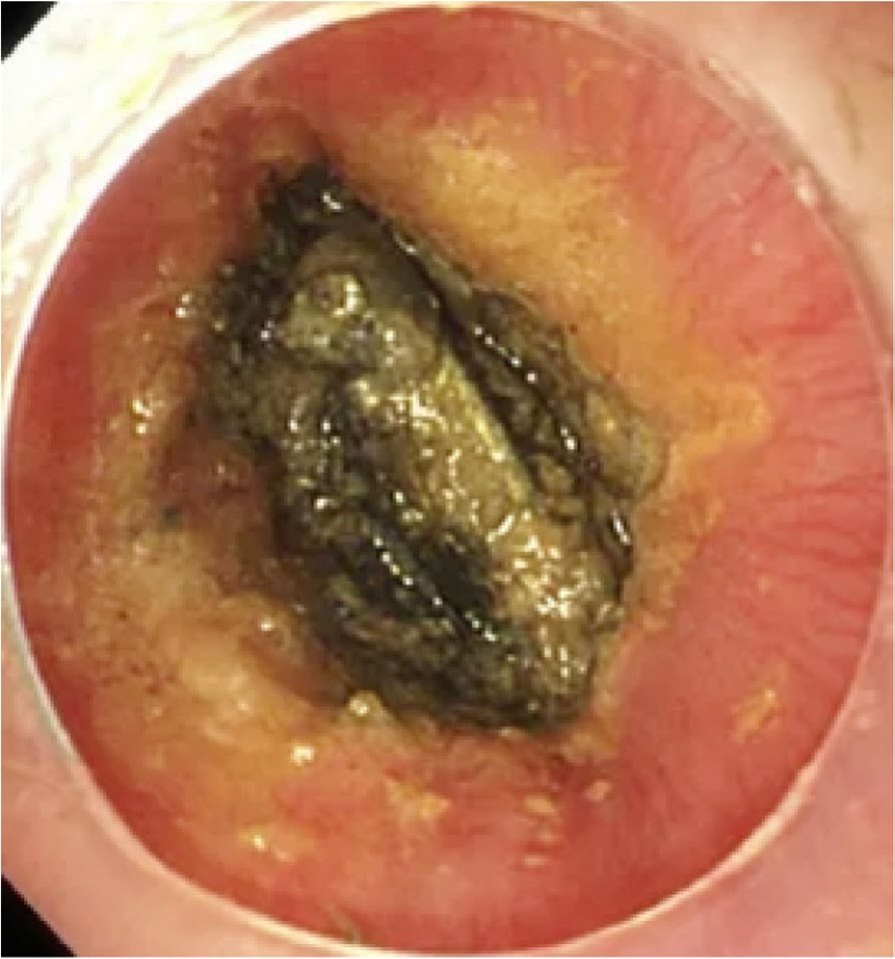
Oesophageal foreign body (button battery) ingestion

**Fig. 4:**
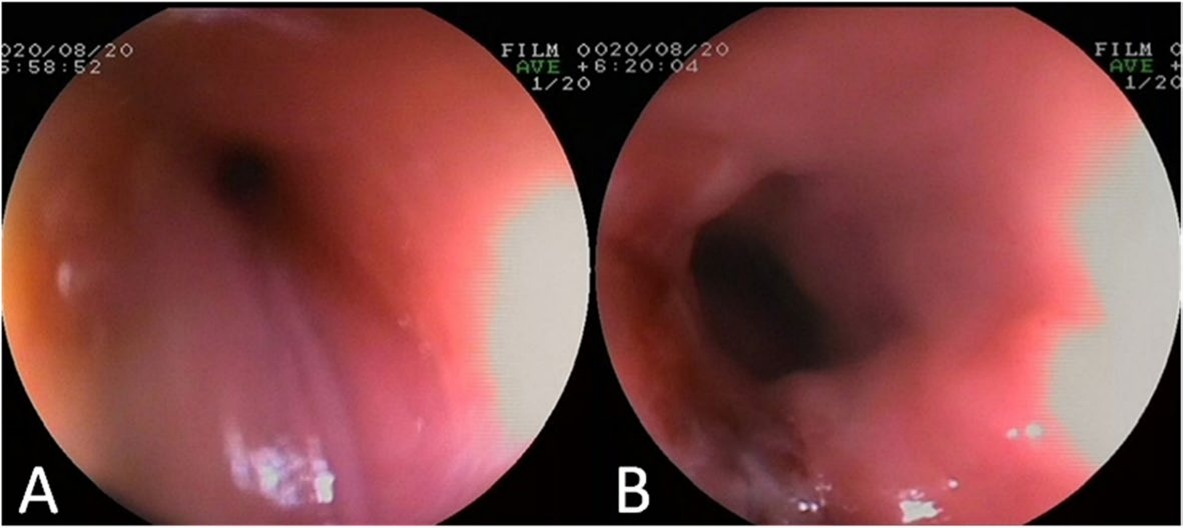
Gastroscopy results (**A** Oesophageal stenosis, **B** oesophageal patency after dilatation)

A total of 558 patients were taken to rigid oesophageal endoscopy under general anaesthesia, and foreign bodies were successfully removed in 525 cases. No FBs were found in 33 cases (possibly due to spontaneous passage), and foreign bodies were pushed into the lower digestive tract during operation in 5 cases (all of which included coins or blunt objects, and FB removal was difficult). Most cases (467,83.7%) underwent emergency endoscopy within 24 hours of diagnosis of an EFB; however, the remaining 91 children, including cases with 86 button batteries, 3 cases with nails, 2 cases with opened pins, and 1 case with a sharp blade, underwent a first-aid fast-track channel endoscopy within half an hour. In addition, we found 51 cases (9.2%) of underlying oesophageal diseases in patients who underwent endoscopy examination, 40 patients had postoesophageal anastomotic stricture or oesophageal atresia, and 11 children had eosinophilic oesophagitis. We have observed that underlying oesophageal diseases lead to a higher incidence of EFBs, and they always present with food impaction and exhibit milder clinical symptoms. Complications occurred in 130 cases (23.3%), and they included mucosal injury (31,5.3%), ulceration (13,2.2%), erosion (86, 15.4%), periesophagitis or periesophageal abscess (15,2.6%), oesophageal stenosis (11,2%), oesophageal perforation (19,3.4%), and tracheoesophageal fistula (5,0.9%). The incidence of oesophageal erosion injury caused by button batteries was 100% (86/86). We can see black and brown corroded oesophageal mucosa reaching deep into the muscle under endoscopy (Fig. 3). Eleven cases had oesophageal stenosis (Fig. 4A), and 5 cases had oesophageal perforation, including 3 cases of tracheoesophageal fistula. All patients with complications were fed by nasogastric tube for 2 weeks after the operation, and active anti-infection, acid inhibition and symptomatic treatment were given to the patient for 1 week. When oesophageal stenosis or perforation was found, CT, MRI or an oesophageal barium meal was used to assess the proximity of the injury to important vascular and airway structures. The reexamination results of 18 cases were normal after 2 weeks of continuous nonoperative management; only 1 case was complicated by oesophageal cicatricial stenosis and cured by balloon oesophageal dilatation 1 month after surgery (Fig. 4B).

### Risk factors related to complications

Complications occurred in 130 of the 558 patients undergoing rigid oesophageal endoscopy. We assessed whether the risk factors were associated with the demographic characteristics using a univariate analysis. Our results showed that EFB complications were not correlated with sex (*p* > 0.05) but correlated with age (χ^2^ = 16.376, *p* = 0.000), duration time of the FB (χ^2^ = 21.813, p = 0.000), FB incarceration location (χ^2^ = 12.719, *p* = 0.002), type of FB (χ^2^ = 339.288, *p* = 0.000) and symptoms (χ^2^ = 46.649, *p* = 0.000). We observed higher incidences of complications in children with younger age, longer duration time, sharp foreign bodies, button batteries, FBs located in the upper oesophagus, and cough or fever.

## Discussion

EFBs are a common emergency in paediatrics. Because children are in the stage of development and exploration, objects are often placed in the oral cavity for play. At the same time, imperfect swallowing function, undererupted teeth, the parents’ lack of awareness of protection and incorrect feeding methods easily lead to accidental swallowing by children. Delayed treatment or improper treatment can cause complications. To reduce the occurrence of dangerous complications, it is important to emphasize the education of parents, early and reasonable diagnosis, and proper treatment.

EFBs are highly prevalent in children under 3 years of age, and our data show that children under 3 years old accounted for 56% of the cases, which was generally similar to previous studies [2, 9]. The common clinical manifestations include vomiting, drooling, feeding refusal, and neck and chest pain. In addition, unlike adults, children may be completely asymptomatic. According to the European Society of Pediatric Gastroenterology, Hepatology, and Nutrition guidelines, drooling and vomiting are among the predominant complaints in children with EFBs [10]; however, drooling and dysphagia were the major discomforts in our study. Not witnessing FB ingestion by caregivers is an important reason for the delay in seeking medical advice [11]; caregivers could not provide a clear history of foreign body ingestion in 137 cases (10.1%). One child ingested a button battery for more than 1 month, which eventually resulted in a tracheoesophageal fistula.

There are many kinds of foreign bodies in the oesophagus; bones were more common in adults, and coins were more relatively common in children [12], which was similar to our results (772 coins, 57%). Children have more access to coins, which are daily circulating items, and are prone to accidental ingestion of coins, but the incidence of ingesting coins is gradually decreasing due to the application of mobile payment in China. Animal bones in food, if accidentally swallowed, are also prone to becoming lodged due to sharp edges. Coins, bones, and button batteries were the three most common FBs in 0- to 3-year-old children. Coins, bones and toys were most common at 3–6 years old, while bones, coins, and food impaction were more likely to be found in children over 6 years old. Previous literature reported that EFBs were located in the upper oesophagus, accounting for 80.5% [13], which is similar to our result (82.6%).

The treatment methods for EFBs include the Foley catheter method, rigid oesophageal endoscopy, digestive endoscopy (flexible oesophageal endoscopy), and Hopkins endoscopy. The use of a Foley catheter to sweep out coins lodged in the oesophagus while the patient is maintained in the Trendelenburg position has been reported. We found a lower success rate when the foreign body stayed longer than 3 days or was located in the middle or lower oesophagus. However, the use of a Foley catheter may lead to concerns about perforation, aspiration, and acute airway obstruction if the operator uses it incorrectly; the only fatal case of the Foley catheter method was reported by Hawkins in 1990, and it was caused by a coin falling into the airway [14]. We did not observe any potential complications in any of the cases, and only 2 children bit their lips due to struggling. Overall spontaneous clearance of coins occurs in approximately 30% of patients, whereas coins in the middle or lower oesophagus may clear before oesophageal endoscopy in as many as 60% of patients [14]. The Foley catheter is a safe, efficient, convenient, and practical method for the treatment of children with coins and other blunt EFBs, and it can significantly reduce pain and be used to avoid unnecessary endoscopy.

Rigid or flexible endoscopy choice does not have certain indications, and some meta-analyses showed no significant difference in the efficacy and safety of rigid and flexible endoscopy in the treatment of oesophageal foreign bodies [15, 16]. However, flexible endoscopy is mostly used in adults under topical anaesthesia, which is beneficial for observing small foreign bodies and performing tissue biopsy. Children with EFBs are more likely to have symptoms of airway obstruction than adults, and rigid oesophageal endoscopy under general anaesthesia can safely protect the airway [17, 18]. Depending on our experience, we strongly advocate rigid endoscopy for children because it allows both the use of optical forceps with a strong grasping ability for EFBs and the positioning of sharp and pointed objects inside the rigid endoscope. We recommend the retrieval net as the first tool for food impaction, and rat tooth retrieval forceps are the best tool for sharp and pointed FBs in the oesophagus.

The factors of complications in EFB patients are complex. Previous studies have shown that sharp foreign bodies and long durations have a higher incidence of complications due to mechanical damage to the mucosa [12, 19]. Our study showed that the age of the patient, the time of foreign body incarceration, the type of foreign body, the location of foreign body incarceration, and the presence of fever or cough were risk factors leading to oesophageal foreign body complications, and the differences were significant. The younger the child is, the sharper the foreign body, the longer the foreign body impaction time, the more severe the mechanical damage to the mucosa, and the higher the probability of complications. First, young children are more vulnerable because the lumen at the entrance of the oesophagus is narrow and the oesophageal muscle layer is weak. Second, a sharp foreign body and button battery may cause oedema and inflammation of the oesophageal mucosa, and long-term compression of the oesophageal mucosa will cause ischaemic necrosis. They are more likely to have fever and cough. Finally, oesophageal endoscopy performed in a narrow and oedematous oesophageal cavity also increases the risk of mechanical damage and mucosal tearing injury. The risk of complications will significantly increase if the duration exceeds 24 hours regardless of the type of foreign body [20]. Most studies suggest that the removal time of oesophageal foreign bodies should be within 24 h or even within 8 h to avoid prolonged mechanical stimulation and cause oesophageal injury [4, 6, 13, 21]. In our study, we found that 51 cases (9.2%) had underlying oesophageal diseases in patients who underwent endoscopy examination, 40 patients had postesophageal anastomotic stricture or oesophageal atresia, and 11 children had eosinophilic oesophagitis. Oesophageal disease should be highly suspected, especially in patients who have been diagnosed with food impaction more than once. Ibrahim [21] investigated underlying oesophageal disease in 12.4% of children with EFBs, and food impaction is a common sign in eosinophilic oesophagitis patients even in the absence of oesophageal stenosis. The endoscopist needs to remove the food bolus by using a grasping device such as a retrieval net instead of pushing the food bolus down to the stomach as a first attempt because such an operation could increase the risk of perforation.

Button batteries have been widely used in electronic products in recent years, and the incidence of button battery ingestion has gradually increased, which can cause chemical corrosion, electrical damage, thermal burns, and physical compression to the oesophageal mucosa. Oesophageal necrosis and even life-threatening complications can occur within 2 hours after accidentally ingesting a button battery [4, 6]. Therefore, the removal of oesophageal button batteries in a timely manner is extremely important. We found that vomiting of yellow–brown foamy secretions is a characteristic symptom in children with oesophageal button battery ingestion, and the characteristic “double ring or halo sign” can also be seen on chest X-ray. An emergency operation should be performed as soon as possible to reduce the corrosion of the oesophageal mucosa. The first-aid fast track channel is conducted within half an hour of patients being diagnosed with oesophageal button batteries. There is no need to wait for fasting and preoperative examinations. We used a large amount of normal saline to wash the oesophageal mucosa after the button battery was removed. All cases were fed a gastric tube and treated with antimicrobial and acid-inhibiting agents after the operation.

Recently, research [22, 23] showed that irrigation with a weakly acidic solution in cadaveric porcine models would help minimize oesophageal damage. The authors found that the use of 0.25% acetic acid after button battery removal could decrease the tissue pH from 12 to 6. Furthermore, they reported that various acidic substances, such as orange juice, honey, and sucralfate, also helped neutralize the pH and reduced the severity of the injury. The authors suggest administering a weakly acidic solution orally immediately after ingestion to help reduce oesophageal injury before emergent endoscopic battery removal. However, it is not safe to give anything by mouth if oesophageal perforation potentially exists, as it could increase the risk of tracheal aspiration with a button battery impacting the oesophagus. A previous study showed that button batteries greater than 20 mm in diameter were responsible for almost all severe injuries [24]. These data suggest that manufacturers should produce smaller button batteries to replace large button batteries and avoid these complications.

This study also has some limitations because it was an observational, retrospective, single-centre study. Our data collection was based on retrospective medical records and endoscopy reports with its inherent limitations of case ascertainment bias. In addition, the data collectors were not blinded to the objective of the study.

## Conclusion

In conclusion, it is essential for public and health care awareness campaigns for education about the hazards of EFBs to avoid ingesting foreign bodies by mistake. The Foley catheter is a safe, efficient, convenient, and practical way to treat children with coins and other blunt EFBs, and it can significantly reduce the pain of children and unnecessary resources, especially when endoscopy is not readily available. We strongly advocate rigid endoscopy for children because it allows both the use of optical forceps with a strong grasping ability for EFBs and the positioning of sharp and pointed objects inside the rigid endoscope. Children found to have EFB impaction can be classified according to the risk factors for complications to rapidly develop a precise treatment plan with individualized differences to reduce the occurrence of complications. According to the current guidelines on early management, it is beneficial to reduce the risk of diagnostic delay and complications.

## Data Availability

The datasets used and/or analyzed during the current study are available from the corresponding author on reasonable request.

## References

[1] Oliva S, Romano C, De Angelis P, Isoldi S, Mantegazza C, Felici E, Dabizzi E, Fava G, Renzo S, Strisciuglio C (2020). Foreign body and caustic ingestions in children: A clinical practice guideline. Digest Liver Dis.

[2] Orsagh-Yentis D, McAdams RJ, Roberts KJ, McKenzie LB. Foreign-Body Ingestions of Young Children Treated in US Emergency Departments: 1995-2015. Pediatrics. 2019;143(5):e20181988. 10.1542/peds.2018-1988 30979810

[3] Gurevich Y, Sahn B, Weinstein T (2018). Foreign body ingestion in pediatric patients. Curr Opin Pediatr.

[4] Mubarak A, Benninga MA, Broekaert I, Dolinsek J, Homan M, Mas E, Miele E, Pienar C, Thapar N, Thomson M (2021). Diagnosis, management, and prevention of button battery ingestion in childhood: a european society for paediatric gastroenterology hepatology and nutrition position paper. J Pediatr Gastroenterol Nutr.

[5] Bekkerman M, Sachdev AH, Andrade J, Twersky Y, Iqbal S (2016). Endoscopic Management of Foreign Bodies in the Gastrointestinal Tract: A Review of the Literature. Gastroenterol Res Pract.

[6] Kramer RE, Lerner DG, Lin T, Manfredi M, Shah M, Stephen TC, Gibbons TE, Pall H, Sahn B, McOmber M (2015). Management of ingested foreign bodies in children: a clinical report of the NASPGHAN Endoscopy Committee. J Pediatr Gastroenterol Nutr.

[7] Gatto A, Capossela L, Ferretti S, Orlandi M, Pansini V, Curatola A, Chiaretti A. Foreign Body Ingestion in Children: Epidemiological, Clinical Features and Outcome in a Third Level Emergency Department. Children (Basel, Switzerland). 2021;8(12):1182. 10.3390/children8121182 PMC8700598 34943378

[8] Eisen GM, Baron TH, Dominitz JA, Faigel DO, Goldstein JL, Johanson JF, Mallery JS, Raddawi HM, Vargo JJ, Waring JP (2002). Guideline for the management of ingested foreign bodies. Gastrointest Endosc.

[9] Boo SJ, Kim HU (2018). Esophageal Foreign Body: Treatment and Complications. Korean J Gastroenterol.

[10] Thomson M, Tringali A, Dumonceau JM, Tavares M, Tabbers MM, Furlano R, Spaander M, Hassan C, Tzvinikos C, Ijsselstijn H (2017). Paediatric gastrointestinal endoscopy: European society for paediatric gastroenterology hepatology and nutrition and European society of gastrointestinal endoscopy guidelines. J Pediatr Gastroenterol Nutr.

[11] Altokhais TI, Al-Saleem A, Gado A, Al-Qahtani A, Al-Bassam A (2017). Esophageal foreign bodies in children: Emphasis on complicated cases. Asian J Surg.

[12] Geng C, Li X, Luo R, Cai L, Lei X, Wang C (2017). Endoscopic management of foreign bodies in the upper gastrointestinal tract: a retrospective study of 1294 cases. Scand J Gastroenterol.

[13] Dipasquale V, Romano C, Iannelli M, Tortora A, Melita G, Ventimiglia M, Pallio S (2022). Managing Pediatric Foreign Body Ingestions: A 10-Year Experience. Pediatr Emerg Care.

[14] Hawkins DB (1990). Removal of blunt foreign bodies from the esophagus. Ann Otol Rhinol Laryngol.

[15] Yang W, Milad D, Wolter NE, Propst EJ, Chan Y (2020). Systematic review of rigid and flexible esophagoscopy for pediatric esophageal foreign bodies. Int J Pediatr Otorhinolaryngol.

[16] Ferrari D, Aiolfi A, Bonitta G, Riva CG, Rausa E, Siboni S, Toti F, Bonavina L (2018). Flexible versus rigid endoscopy in the management of esophageal foreign body impaction: systematic review and meta-analysis. World J Emerg Surg.

[17] Ikenberry SO, Jue TL, Anderson MA, Appalaneni V, Banerjee S, Ben-Menachem T, Decker GA, Fanelli RD, Fisher LR, Fukami N (2011). Management of ingested foreign bodies and food impactions. Gastrointest Endosc.

[18] Ergun E, Ates U, Gollu G, Bahadir K, Yagmurlu A, Cakmak M, Aktug T, Dindar H, Bingol-Kologlu M. An algorithm for retrieval tools in foreign body ingestion and food impaction in children. Dis Esophagus. 2021;34(1):doaa051. 10.1093/dote/doaa051 32519749

[19] Hong KH, Kim YJ, Kim JH, Chun SW, Kim HM, Cho JH (2015). Risk factors for complications associated with upper gastrointestinal foreign bodies. World J Gastroenterol.

[20] Wu WT, Chiu CT, Kuo CJ, Lin CJ, Chu YY, Tsou YK, Su MY (2011). Endoscopic management of suspected esophageal foreign body in adults. Dis Esophagus.

[21] Ibrahim AH, Andijani A, Abdulshakour M, Algain S, Thamrah AA, Ali MM, Marwah H, Aldaher A, Bashir S, Alsaleem B (2021). What do saudi children ingest?: a 10-year retrospective analysis of ingested foreign bodies from a tertiary care center. Pediatric Emergency Care.

[22] Jatana KR, Rhoades K, Milkovich S, Jacobs IN (2017). Basic mechanism of button battery ingestion injuries and novel mitigation strategies after diagnosis and removal. Laryngoscope.

[23] Anfang RR, Jatana KR, Linn RL, Rhoades K, Fry J, Jacobs IN (2019). pH-neutralizing esophageal irrigations as a novel mitigation strategy for button battery injury. Laryngoscope.

[24] Pugmire BS, Lin TK, Pentiuk S, de Alarcon A, Hart CK, Trout AT (2017). Imaging button battery ingestions and insertions in children: a 15-year single-center review. Pediatric Radiol.

